# Genetic variants and phenotypic data curated for the CAGI6 intellectual disability panel challenge

**DOI:** 10.1007/s00439-025-02733-1

**Published:** 2025-02-28

**Authors:** Maria Cristina Aspromonte, Alessio Del Conte, Roberta Polli, Demetrio Baldo, Francesco Benedicenti, Elisa Bettella, Stefania Bigoni, Stefania Boni, Claudia Ciaccio, Stefano D’Arrigo, Ilaria Donati, Elisa Granocchio, Isabella Mammi, Donatella Milani, Susanna Negrin, Margherita Nosadini, Fiorenza Soli, Franco Stanzial, Licia Turolla, Damiano Piovesan, Silvio C. E. Tosatto, Alessandra Murgia, Emanuela Leonardi

**Affiliations:** 1 Department of Biomedical Sciences, University of Padova, Padua, Italy; 2 Molecular Genetics of Neurodevelopment, Department of Woman and Child Health, University of Padova, Padua, Italy; 3 Fondazione Istituto Di Ricerca Pediatrica (IRP), Città Della Speranza, Padua, Italy; 4 Medical Genetics Unit, Treviso Hospital, Treviso, Italy; 5 Genetic Counseling Service, Regional Hospital of Bolzano, Bolzano, Italy; 6 Medical Genetics Unit, Ferrara University Hospital, Ferrara, Italy; 7 Medical Genetics Unit, S. Martino Hospital, Belluno, Italy; 8 Department of Pediatric Neurosciences, Fondazione IRCCS Istituto Neurologico “Carlo Besta”, Milan, Italy; 9 Unit of Medical Genetics, AUSL Romagna, Cesena, Italy; 10 Medical Genetics Unit, Mirano Hospital, Venice, Italy; 11 Fondazione IRCCS, Ca’ Granda Ospedale Maggiore Policlinico, Milan, Italy; 12 Scientific Institute, IRCCS E. Medea, dipartimento/Unità Operativa Conegliano, Treviso, Italy; 13 Paediatric Neurology and Neurophysiology Unit, Department of Women’s and Children’s Health, University Hospital of Padova, Padua, Italy; 14 Genetic Unit, UOM Patologia Clinica, S. Chiara Hospital of Trento, Trento, Italy; 15 CNR IBIOM, Bari, Italy

## Abstract

Neurodevelopmental disorders (NDDs) are common conditions including clinically diverse and genetically heterogeneous diseases, such as intellectual disability, autism spectrum disorders, and epilepsy. The intricate genetic underpinnings of NDDs pose a formidable challenge, given their multifaceted genetic architecture and heterogeneous clinical presentations. This work delves into the intricate interplay between genetic variants and phenotypic manifestations in neurodevelopmental disorders, presenting a dataset curated for the Critical Assessment of Genome Interpretation (CAGI6) ID Panel Challenge. The CAGI6 competition serves as a platform for evaluating the efficacy of computational methods in predicting phenotypic outcomes from genetic data. In this study, a targeted gene panel sequencing has been used to investigate the genetic causes of NDDs in a cohort of 415 paediatric patients. We identified 60 pathogenic and 49 likely pathogenic variants in 102 individuals that accounted for 25% of NDD cases in the cohort. The most mutated genes were *ANKRD11, MECP2, ARID1B, ASH1L, CHD8, KDM5C, MED12* and *PTCHD*1 The majority of pathogenic variants were de novo, with some inherited from mildly affected parents. Loss-of-function variants were the most common type of pathogenic variant. In silico analysis tools were used to assess the potential impact of variants on splicing and structural/functional effects of missense variants. The study highlights the challenges in variant interpretation especially in cases with atypical phenotypic manifestations. Overall, this study provides valuable insights into the genetic causes of NDDs and emphasises the importance of understanding the underlying genetic factors for accurate diagnosis, and intervention development in neurodevelopmental conditions.

**Supplementary Information:**

The online version contains supplementary material available at 10.1007/s00439-025-02733-1.

## Introduction

The field of neurodevelopmental disorders (NDDs) encompasses a diverse group of conditions characterised by impairments in cognitive, motor, and social functions that arise early in development. The study of the genetic causes of NDDs is complicated by its extensive genetic and phenotypic heterogeneity, in addition to a high degree of comorbidity (Morris-Rosendahl and Crocq 2020). The increased co-occurrence of these disorders, which include (among others) intellectual disability, autism spectrum disorder, language/speech disorders and epilepsy, suggested shared genetic aetiology among NDDs (Jensen and Girirajan 2017). Multiple genetic mechanisms can result in overlapping NDD phenotypes and, simultaneously, a single genetic mechanism can result in a range of phenotypic outcomes (Parenti et al. 2020). Understanding the underlying genetic causes of NDDs is crucial for accurate diagnosis, prognosis, and the development of targeted interventions.

Technological advances in genomics have enabled the identification of a huge amount of rare genetic data from both general population cohorts (e.g. Genome Aggregation Database) (Karczewski et al. 2020) as well as particular diseases (e.g., Autism Spectrum Disorders) (Feliciano et al. 2019; Zhou et al. 2022). The aggregation and sharing of phenotypic and genetic data through public or restricted-access databases and international collaborative efforts has led to a remarkable progress in the understanding of the genetic architecture and neurobiology underlying NDDs (Cousin et al. 2022; Fu et al. 2022; Gehin et al. 2023; Satterstrom et al. 2020). Many studies highlighted the contribution of de novo variants in the NDD liability model (Coe et al. 2019; Iossifov et al. 2014; Kaplanis et al. 2020; Krumm et al. 2015; Satterstrom et al. 2020). On the other hand, many NDD cases appear to result from the effects of a polygenic inheritance mode, in which the burden of *de nov*o, inherited rare and common variants in multiple genes all contribute to the phenotype (Fu et al. 2022; Grove et al. 2019; Zhou et al. 2022). Rare genetic variations, particularly truncating variants, have played a crucial role in the characterization of hundreds of NDD-related genes. Although the presence of missense variants in disease genes can be indicative of their deleteriousness, the significance of many missense variants remains uncertain due to position-specific effects.

Both this challenge and the previous one (Aspromonte et al. 2019) were conducted as part of the Critical Assessment of Genome Interpretation (CAGI) experiment (Critical Assessment of Genome Interpretation Consortium 2024). CAGI is a worldwide blind test to assess the accuracy of computational methods to predict the phenotypic impact of genomic variations and guide future research. The Neurodevelopmental Disorders Lab (NDDs Lab at Dept. of Woman and Child Health, Padova University Hospital, Italy) and the BioComputingUP group (Dept. of Biomedical Sciences, University of Padova, Italy) participated as data providers and assessors for the Intellectual Disability (ID) panel challenge in CAGI5 and CAGI6 (Carraro et al. 2019; Aspromonte et al. 2025). In both editions, the challenge was to predict pathogenic mutations and presence or absence of seven clinical traits (intellectual disability, autism spectrum disorder, epilepsy, macrocephaly, microcephaly, hypotonia, ataxia) in each patient from sequencing data in a 74 gene panel. Predictors are provided with VCF files containing sequence data to identify the causal variant(s) responsible for each of the seven phenotypic traits. The assessors then evaluate the anonymized predictions, ensuring a high level of rigor and objectivity.

In a previous study, we presented the findings obtained with the application of a targeted gene panel sequencing in 150 NDD individuals (Aspromonte et al. 2019). Overall, this study provided a valuable guide for the interpretation of genetic variants, based on expert knowledge related to the disease phenotype and gene functions. The description of diagnosed cases highlighted the critical steps of variant interpretation in the clinical diagnostic context of neurodevelopmental conditions.

Our data have been used as the “ID panel challenge” dataset to assess computational approaches aiming to predict comorbid phenotypes from genetic variants in a subset of NDD genes (Carraro et al. 2019). The participation of the four predictor groups in this experiment provided a basis for the development of improved methods as well as for designed and automated use in clinical settings (J. Chen 2019; Critical Assessment of Genome Interpretation Consortium 2024).

In this study we present the genetic and phenotypic data as well as the findings of the same 74 gene panel sequencing obtained on a cohort of 415 new individuals with NDDs. This dataset has been used in a new “ID panel challenge” of the sixth edition of CAGI (CAGI6). Like in the previous study (Aspromonte et al. 2019), here we describe the diagnosed cases that could explain the difficulties in variant interpretation and in predicting the associated phenotypes.

## Materials and methods

### Patient selection

The 415 cases of this cohort were selected from those referred to the NDDs Lab, from March 2017 to June 2019, for gene panel testing. Clinical data were collected by the referring clinicians/geneticists from 17 Italian public hospitals with a standardised clinical record describing family history, clinical phenotype, previous genetics, metabolics and neurophysiological investigations. Table 1 summarises the clinical data of the patients, while Supplementary Table S1 reports for each of 415 patients the presence of ID (HP:0001249), Autism Spectrum Disorders (ASD) (HP:0000729), epilepsy (HP:0001250), microcephaly (HP:0000252) or macrocephaly (HP:0000256), hypotonia (HP:0001252), and ataxia (HP:0001251). Each phenotype was intended to follow the criteria defined by the corresponding Human Phenotype Ontology (HPO) terms (Köhler et al. 2017). Written informed consent was obtained from the patient’s parents or legal representative.

**Table 1:** Description of the cohort of 415 individuals enrolled for the CAGI6 ID panel challenge. The table includes demographic information, clinical features as well as results from genetic and neurological investigations

Features	Patients (*n* = 415)
Gender	
Female	147 (35%)
Male	268 (65%)
*Total*	415
Age (year old, at diagnosis)	
[0–10]	277 (67%)
[11–20]	122 (29%)
> 20	16 (4%)
Familial history	
Sporadic	320 (77%)
Familial	95 (23%)
Sib pair	24 (6%)
X-linked	2 (0.5%)
Intellectual disability	
Total reported	352 (84.8%)
Mild	109 (31%)
Mild/moderate, moderate	100 (28%)
Severe	70 (20%)
Not evaluated	73 (21%)
Not reported	53 (12.8%)
Normal cognitive function	10 (2.4%)
Comorbidity	
ASD (autistic features)	205 (49%)
ASD not reported	62 (15%)
Epilepsy	84 (20%)
Hypotonia	71 (17%)
Ataxia	30 (7%)
Microcephaly	45 (11%)
Macrocephaly	40 (10%)
Other investigation	
aCGH	338 (82%)
X-Fragile	217 (52%)
Other genetic tests (single genes, gene panels)	84 (20%)
EEG anomaly	99 (24%)
MRI anomaly	84 (20%)
aCGH	338 (82%)

*ASD* autism spectrum disorder, *aCGH* array‐comparative genomic hybridization, *EEG* electroencephalogram, *MRI* magnetic resonance imaging, *X-linked* variants in genes located on X-chromosome

### Gene panel sequencing and data analysis

We sequenced 74 genes in DNA samples from 415 paediatric patients with NDDs. The sequencing was performed using the Ion Torrent platform at the Laboratory of Padova as previously described in (Aspromonte et al. 2019). We designed a cost-effective targeted panel using Ion AmpliSeq™ Designer, to amplify all exons and flanking regions (10 bp) of 74 genes associated with ID and ASD comorbidities (see Supplementary Table 3). To create a variant in-house database, we developed a pipeline to annotate the variants using ANNOVAR (Wang et al. 2010; Yang and Wang 2015) and calculate the frequency of each variant within our dataset. The variants were filtered based on sequencing parameters, frequency in the general population (eg. gnomAD v4.1.0) (S. Chen et al. 2022) and in the in-house database, and pathogenicity predictions. Segregation analysis was conducted using Sanger sequencing when DNA samples from relatives were available. For de novo variants, paternity and maternity were confirmed as described before (Aspromonte et al. 2019). For variants detected on X-linked genes, the X-inactivation pattern was assessed on the human androgen receptor (AR) gene locus. When possible, intron and splicing variants were analysed using mRNA extracted from patient peripheral blood leukocytes through reverse transcription of cDNA. Variant interpretation followed the criteria outlined by the American College of Medical Genetics and Genomics (ACMG) (Richards et al. 2008) rich. For the final classification of the identified variants we used the same workflow used for CAGI5 challenge, a detailed description has been published in Aspromonte et al. (Aspromonte et al. 2019).

### In silico analysis of the variants

The potential impact on splicing signals has been evaluated by Human Splicing Finder (HSF) (Desmet et al. 2009). Pathogenicity of missense variants has been predicted using computational methods provided by ANNOVAR (Yang & Wang 2015) and Combined Annotation Dependent Depletion (CADD) (Rentzsch et al. 2019). Conservation of mutated positions has been evaluated with GERP + + (Davydov et al. 2010). Structural effects of missense variants for *GRIN2A*, *SLC6A1*, *ANKRD11*, *IQSEC2* have been evaluated using the structures deposited in the Protein Data Bank database (https://www.rcsb.org/) (Berman et al. 2000; Burley et al. 2023) (PDB codes: 6IRA; 7SK2; 6FAE) or the AlphaFold models (Jumper et al. 2021). Variants were mapped and visualised on their respective structures using Pymol Molecular Graphics System Version (Schrödinger, LLC, 2021).

Protein sequences, retrieved from the UniProtKB database (UniProt Consortium, 2023), have been aligned with MAFFT (Katoh and Standley 2013) and visualised with Jalview. Secondary structure and amino-acid enrichment has been predicted by FELLS (Piovesan et al. 2017), while the MobiDB (Piovesan et al. 2025), DisProt (Aspromonte et al. 2023) and ELM (Kumar et al., 2021) resources have been used to predict or retrieve information of intrinsically disordered regions (IDRs) and functional linear motifs, respectively.

## Results

### Cohort description

A total of 415 new paediatric patients with NDDs, never described before, were included in this study. The cohort primarily consisted of 268 males (65%) and patients under 20 years of age (96%) (see Table 1). 320 (77%) were sporadic cases, however a family history for NDDs was reported in 95 (23%) cases, with affected siblings in 6%. Intellectual Disability (ID) was the most common clinical feature, reported in 84.8% of cases, with a higher proportion of mild to moderate forms. While only 20% of the cases were reported with a severe intellectual impairment. Intellectual Quotient (IQ) level was not evaluated in 73 cases with ID (21%). Only ten patients (2.4%) presented a normal IQ, while information regarding cognitive impairment was not reported in 53 (12.8%) children (see Table 1). ASD was reported in 49% of patients, with 40% of them also having ID. Epilepsy, ataxia, microcephaly, and macrocephaly were fewer common phenotypes. At least one clinical trait was present in each patient, and complete information for all seven phenotypic traits was available for 47% of cases. Based on Table 1, we can identify the most and least common combinations of clinical traits in our cohort. The co-occurrence of ID and ASD is the most frequent comorbidity, observed in 161 patients (38.8%). Among cases with more than two phenotypic traits, the most common complex conditions include ID, ASD, and Epilepsy in 33 patients (7.9%), or ID, ASD, and Hypotonia in 23 patients (5.5%). Isolated ASD is rare, with only 5 patients (1.2%). A single patient (UniPD_0146) has the most complex phenotype, exhibiting ID, ASD, epilepsy, microcephaly and muscle tone abnormalities (Hypotonia).

Despite various genetic analyses, the majority of patients remained undiagnosed. However, array CGH revealed copy number variants (CNVs) classified as likely benign or of uncertain significance in 79 cases. Two patients with chromosomal alterations, XXX and XXY karyotype, were diagnosed with Trisomy X and Klinefelter syndrome, respectively (UniPD_0267 and UniPD_0284).

### Mutated genes and variants type

**Fig. 1:**
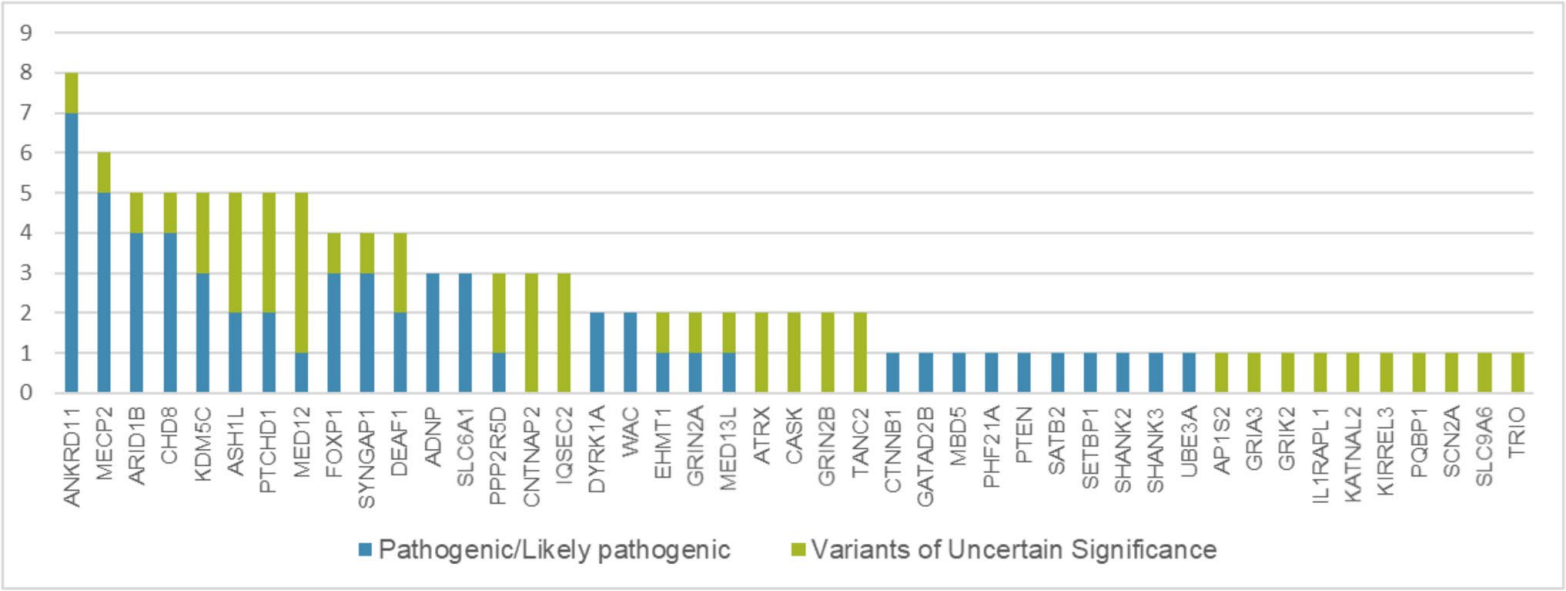
Number of pathogenic/ likely pathogenic (Blu bars) and Variants of Uncertain Significance (green bars) identified in the cohort of 415 individuals. The *ANKRD11* gene exhibits the highest number of pathogenic/likely pathogenic variants, while several other genes show a mix of both variant categories

**Fig. 2:**
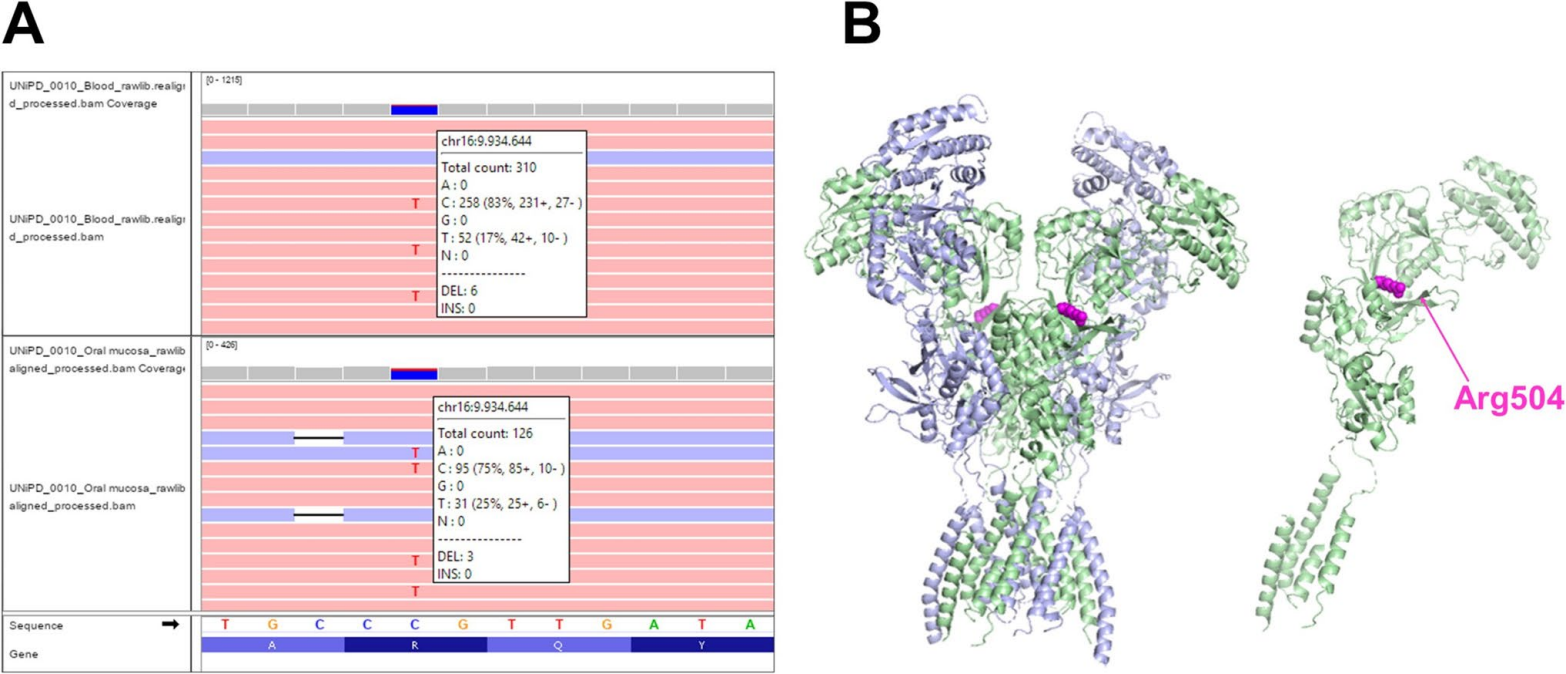
Mosaic variant in the *GRIN2A* gene. **A** IGV visualization of the mosaic missense variant detected in *GRIN2A*, analyzed using the Ion Torrent PGM platform on DNA extracted from a blood sample (top) and oral mucosa cells (bottom). **B** Structure of the human GluN1/GluN2A NMDA receptor in the glutamate/glycine-bound state (left) and GluN2A NMDA receptor molecule (right) (PDB code: 6IRA). The Arginine 504 residue is indicated as a purple sphere

**Fig. 3:**
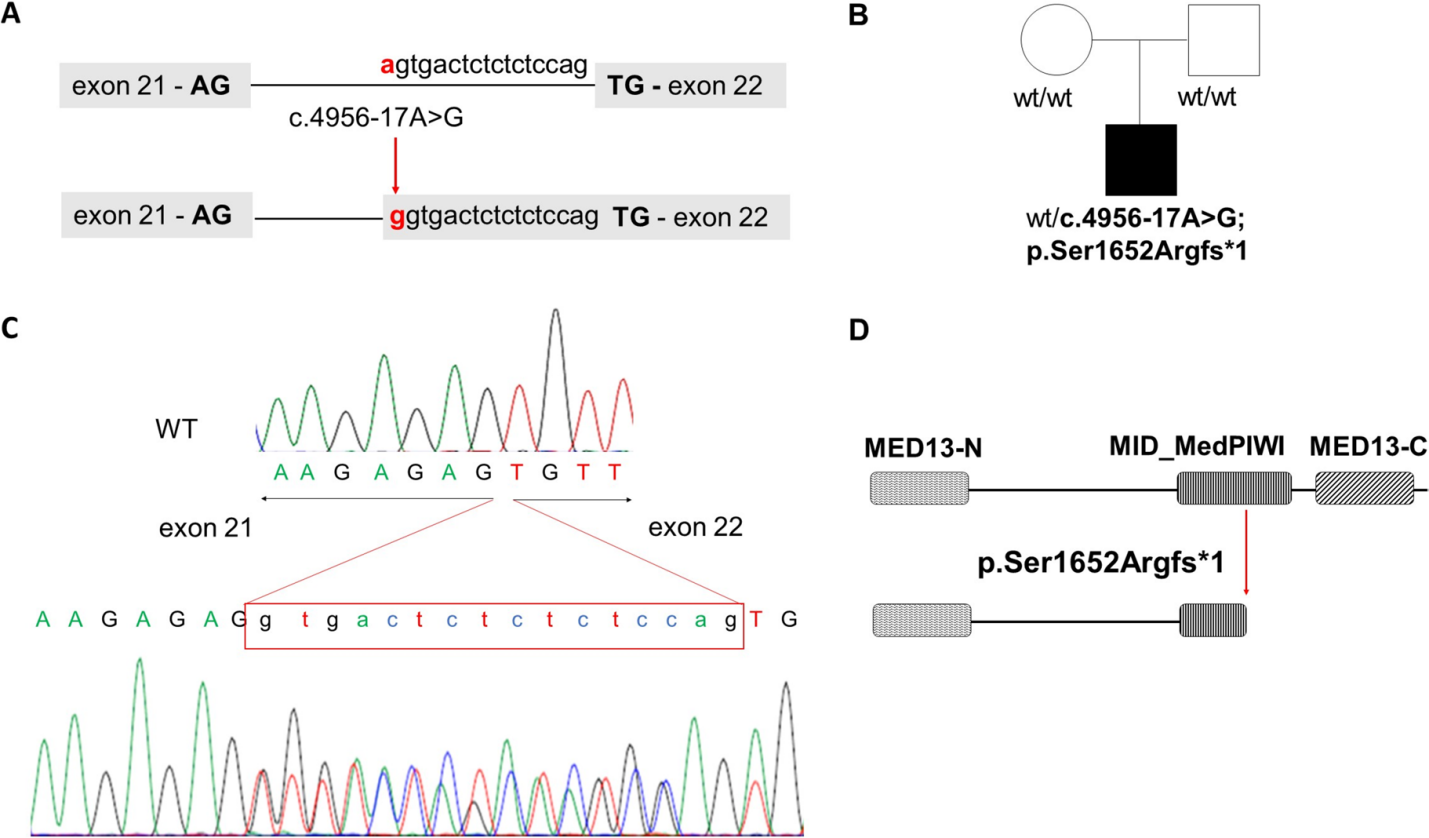
De novo intronic variant c.4956-17A in *MED13L*. **A** Localisation of *GRIN2A* variant in the genomic sequence and its predicted effect on splicing process; **B** Transcript analysis of the intronic variant in *MED13L* performed for the control sample (top) and patient’s sample (bottom). The intronic variant generates a new Acceptor splicing site leading to the inclusion of 16 intronic nucleotides (intron 21) into exon 22; **C** Pedigree showing the absence of intronic variant in healthy parents; **D** The predicted premature truncated protein, p.Ser1652Argfs*1, will miss the MID domain of the Med PIWI module, the core globular domain of MED13 protein

The low-cost targeted gene-panel designed for ID/ASD enabled us to obtain high-quality sequencing data, with a coverage > 100 × in the targeted regions. In our cohort we identified 60 pathogenic/likely pathogenic (P/LP) variants (see Table 2) and 49 Variants of Uncertain Significance (VUS) (see Table 3). The reported variants map to 45 different genes (see Fig. 1). For 16 genes, variants were detected in more than two patients. *ANKRD11* was the most frequently mutated gene, with seven detected variants. *MECP2* variants were found in six individuals, and *ARID1B, ASH1L, CHD8, KDM5C, MED12* and *PTCHD*1 each had variants in five cases. Out of the 60 (14.5%) patients who received a definitive genetic diagnosis, 18 had a reported family history, 17 patients were sporadic, while family information was unavailable for 25 patients. Most of the pathogenic variants (n = 44; 73%) were de novo, three inherited from mildly affected parents, three were not inherited from the single available parent, and for 16.6% (n = 10) of the variants, inheritance could not be assessed (see Table 1). The majority of pathogenic variants, 36 out of 60, are loss-of-function (LoF) variants. Specifically, 16 introduce a novel stop codon, 20 are frameshift insertions or deletions, and 22 variants are missense. Among them we identified the somatic mosaicism variant p.(Arg504Gln) in *GRIN2A*. Targeted next generation sequencing on the proband’s blood sample and oral mucosa cells allowed for the estimation of a mosaicism of 25% (see Fig. 2). Additionally, we identified a novel de novo non-frameshift substitution variant in the *DEAF1* gene p.(Pro237_Thr238delinsSerSer) and one de novo deep intronic variant (c.4956-17A > G) in *MED13L* which impacts the splicing mechanism (see Fig. 3). The majority of P and LP variants were identified in patients whose clinical phenotype matched the mutated gene. In other individuals, we observed clinical features that were atypical for the mutated genes (see Table 4).

**Table 2:** Pathogenic/Likely Pathogenic variants found in 415 paediatric patients

P	S	GENE	INH	ZYG	VFS	FH	Type	Ref Seq	Variant	dbSNP	Clinvar	MAF	CP	CADD	GERP
0107	F	*ADNP*	AD	HT	DN	–	MIS	NM_001282531.3	c.2473G > C p.(Gly825Arg)	–	–	–	8	25	5.1
0129	M	*ADNP*	AD	HT	–	–	FS	NM_001282531.3	c.2232_2236del p.(Glu744AspfsTer2)	–	–	–	–		
0072	M	*ADNP*	AD	HT	DN	–	FS	NM_001282531.3	c.539_542del p.(Val180GlyfsTer17)	rs1057518345	P	–	–		
0291	M	*ANKRD11*	AD	HT	DN	–	MIS	NM_013275.6	c.7606C > T p.(Arg2536Trp)	rs2151701893	P/LP	-	9	29	1.7
0256	M	*ANKRD11*	AD	HT	DN	–	FS	NM_013275.6	c.5973_5997del p.(Lys1992SerfsTer87)	–	–	–	–		
0205	F	*ANKRD11*	AD	HT	DN	–	FS	NM_013275.6	c.4396_4397del p.(Arg1466GlyfsTer87)	–	–	–	–		
0171	F	*ANKRD11*	AD	HT	–	–	STOP	NM_013275.6	c.2446G > T p.(Glu816Ter)	–	–	–	–	37	5.7
0212	F	*ANKRD11*	AD	HT	DN	–	FS	NM_013275.6	c.2165_2166del p.(Lys722ArgfsTer19)	–	–	–	–		
0330	F	*ANKRD11*	AD	HT	Absent in healthy sister	–	FS	NM_013275.6	c.1903_1907del p.(Lys635GlnfsTer26)	rs886041125	P	–	–		
0242	M	*ANKRD11*	AD	HT	DN	–	FS	NM_013275.6	c.281_284del p.(Ala94GlyfsTer29)	–	–	–	–		
0142	F	*ARID1B*	AD	HT	–	–	STOP	NM_001374828.1	c.1996C > T p.(Gln666Ter)	rs1554265250	–	–	–	40	5.5
0105	F	*ARID1B*	AD	HT	DN	–	STOP	NM_001374828.1	c.3673C > T p.(Arg1225Ter)	rs387907141	P	–	–	40	5.0
0408	F	*ARID1B*	AD	HT	DN	–	FS	NM_001374828.1	c.4964_4974del p.(Ile1655ThrfsTer100)	–	–	–	–		
0093	M	*ARID1B*	AD	HT	DN	–	MIS	NM_001374828.1	c.6775 T > C p.(Ser2259Pro)	rs1057521854	LP	-	7	27	5.5
0141	M	*ASH1L*	AD	HT	DN	–	MIS	NM_018489.3	c.3893 T > G p.(Leu1298Arg)	–	–	–	11	26	4.9
0337	M	*ASH1L*	AD	HT	DN	–	MIS	NM_018489.3	c.3179A > G p.(Asn1060Ser)	rs1665817487	–	–	6	23	5.1
0390	M	*CHD8*	AD	HT	–	–	FS	NM_001170629.2	c.7148del p.(Pro2383GlnfsTer47)	–	–	–	–		
0393	F	*CHD8*	AD	HT	DN	–	MIS	NM_001170629.2	c.6997C > T p.(Arg2333Cys)	rs1887539639	-	–	6	32	5.4
0336	F	*CHD8*	AD	HT	DN	–	MIS	NM_001170629.2	c.2282G > T p.(Trp761Leu)		-	–	11	29	5.0
0062	M	*CHD8*	AD	HT	DN	–	STOP	NM_001170629.2	c.1174C > T p.(Gln392Ter)	rs1555318204	–	–	–	36	5.4
0246	M	*CTNNB1*	AD	HT	–	–	FS	NM_001904.4	c.160dup p.(Glu54GlyfsTer12)	–	–	–	–		
0395	F	*DEAF1*	AD/AR	HT	DN	–	MIS	NM_021008.4	c.782G > C p.(Arg261Pro)	–	–	–	11	25	4.5
0172	F	*DEAF1*	AD/AR	HT	DN	–	nonFS	NM_021008.4	c.709_712delinsTCCT p.(Pro237_Thr238delinsSerSer)	–	–	–	–		
0362	M	*DYRK1A*	AD	HT	DN	–	STOP	NM_001347721.2	c.427G > T p.(Gly143Ter)	rs1463551651	–	–	–	37	5.2
0331	F	*DYRK1A*	AD	HT	DN	–	STOP	NM_001347721.2	c.664C > T p.(Arg222Ter)	rs780441716	P	–	–		5.6
0112	M	*EHMT1*	AD	HT	DN	–	MIS	NM_024757.5	c.3186C > G p.(Cys1062Trp)	–	–	–	11	23	0.1
0399	M	*FOXP1*	AD	HT	DN	–	STOP	NM_001349338.3	c.1630C > T p.(Arg544Ter)	–	P	–	–	38	4.0
0351	M	*FOXP1*	AD	HT	DN	–	FS	NM_001349338.3	c.1590dup p.(Gly531ArgfsTer8)	–	–	–	–		
0407	F	*FOXP1*	AD	HT	DN	–	STOP	NM_001349338.3	c.1526G > A p.(Trp509Ter)	rs780157776	–	–	–	47	5.9
0286	M	*GATAD2B*	AD	HT	DN	–	MIS	NM_020699.4	c.922 T > G p.(Cys308Gly)	–	–	–	3	23	5.1
0010	F	*GRIN2A*	AD	MOS	DN	–	MIS	NM_001134407.3	c.1511G > A p.(Arg504Gln)	rs1331671132	VUS	–	6	24	5.3
0110	F	*KDM5C*	XL	HT	DN	–	MIS	NM_004187.5	c.3794 T > C p.(Leu1265Pro)	–	-	–	9	27	4.8
0243	M	*KDM5C*	XL	HE	MAT	–	STOP	NM_004187.5	c.2851C > T p.(Arg951Ter)	rs1556837277	P	–	–	36	3.8
0366	F	*KDM5C*	XL	HT	DN	–	MIS	NM_004187.5	c.1795C > T p.(Arg599Cys)	rs1556842184	LP	–	9	29	5.7
0388	M	*MBD5*	AD	HT	DN	–	FS	NM_001378120.1	c.24del p.(Asp8GlufsTer75)	–	–	–	–		
0140	F	*MECP2*	XL	HT	DN	–	STOP	NM_004992.4	c.808C > T p.(Arg270Ter)	rs61750240	P	–	–	37	
0267	F	*MECP2*	XL	XXX	–	–	STOP	NM_004992.4	c.808C > T p.(Arg270Ter)	rs61750240	P	–	–	37	3.7
0411	F	*MECP2*	XL	HT	–	–	STOP	NM_004992.4	c.763C > T p.(Arg255Ter)	rs61749721	P	–	–	38	3.5
0118	F	*MECP2*	XL	HT	–	–	MIS	NM_004992.4	c.473C > T p.(Thr158Met)	rs28934906	P/LP	–	11	27	5.5
0081	F	*MECP2*	XL	HT	–	–	MIS	NM_004992.4	c.316C > T p.(Arg106Trp)	rs28934907	P/LP	–	12	26	2.8
0210	M	*MED12*	XLD	HE	–	+	MIS	NM_005120.3	c.4147G > A p.(Ala1383Thr)	rs863223696	P	–	9	29	4.4
0383	M	*MED13L*	AD	HT	DN	–	SP	NM_015335.5	c.4956-17A > G p.Ser1652Argfs*1	–	–	–	–		
0014	M	*PHF21A*	AD	HT	DN	-	FS	NM_001352027.3	c.1032_1035del p.(Thr345ArgfsTer28)	rs2092369866	P	–	–		
0056	M	*PPP2R5D*	AD	HT	DN	–	MIS	NM_006245.4	c.598G > A p.(Glu200Lys)	rs863225079	P/LP	–	9	32	5.7
0169	F	*PTCHD1*	XL	HT	DN	–	MIS	NM_173495.3	c.2072G > A p.(Arg691Gln)	rs1569143368	–	–	5	23	5.3
0104	M	*PTCHD1*	XL	HE	MAT	+	STOP	NM_173495.3	c.2289C > A p.(Tyr763Ter)	–	–	–	–	35	3.4
0109	M	*PTEN*	AD	HT	DN	–	MIS	NM_000314.8	c.103A > G p.(Met35Val)	rs876659443	P	–	12	25	5.2
0309	M	*SATB2*	AD	HT	DN	–	FS	NM_001172509.2	c.1105_1106insT p.(Arg369MetfsTer3)	–	–	–	–		
0240	M	*SETBP1*	AD	HT	DN	-	STOP	NM_015559.3	c.1765C > T p.(Arg589Ter)	rs1568235086	P/LP	–	–	39	6.1
0418	F	*SHANK2*	AD	HT	DN	–	FS	NM_133266.5	c.2989_3004del p.(Pro997SerfsTer38)	–	–	–	–		
0333	F	*SHANK3*	AD	HT	DN	–	FS	NM_0033517.1	c.4637_4638del p.(Phe1563GlnfsTer5)	–	–	–	–		
0114	M	*SLC6A1*	AD	HT	NOT MAT	–	MIS	NM_003042.4	c.855G > T p.(Trp285Cys)	–	–	–	11	31	4.9
0047	M	*SLC6A1*	AD	HT	DN	–	MIS	NM_003042.4	c.919G > A p.(Gly307Arg)	rs1553689696	P/LP	–	11	26	4.8
0034	M	*SLC6A1*	AD	HT	MAT	+	MIS	NM_003042.4	c.1084G > A p.(Gly362Arg)	rs1131691302	LP	–	11	25	4.9
0299	F	*SYNGAP1*	AD	HT	DN	–	FS	NM_006772.3	c.431_434del p.(Thr144SerfsTer29)	–	–	–	–		
0170	M	*SYNGAP1*	AD	HT	DN	–	FS	NM_006772.3	c.2473_2474dup p.(Asp826ArgfsTer11)	–	–	–	–		
0288	F	*SYNGAP1*	AD	HT	DN	–	FS	NM_006772.3	c.3778_3779del p.(Lys1260GlufsTer22)	–	–	–	–		
0200	F	*UBE3A*	AD	HT	DN	–	FS	NM_130839.5	c.2571_2574dup p.(Leu859GlufsTer23)	–	–	–	–		
0391	M	*WAC*	AD	HT	DN	–	STOP	NM_016628.5	c.139C > T p.(Arg47Ter)	rs368543869	P	-	-	36	3.7
0148	F	*WAC*	AD	HT	NOT PAT	–	STOP	NM_016628.5	c.374C > A p.(Ser125Ter)	rs864321692	P	–	–	38	5.8

*P* patient code, *S* sex, *INH* mode of inheritance, *ZYG* zygosity, *VFS* Variant Family Segregation, *FH* familial history, *Ref Seq* reference sequence, *MAF* minor allele frequency, CP. consensus prediction among 12 computational tools provided by Annovar, *CADD* Combined Annotation Dependent Depletion score, *GERP* genomic evolutionary rate profiling score, *M* Male, *F* Female, *AD* autosomal dominant, *AR* autosomal recessive, *XL* X-linked, *HT* heterozygous, *HE* hemizygous, *MOS* mosaicism, *DN* de novo, *MAT* maternal, *PAT* paternal, *MIS* missense, *FS* frameshift, *P* pathogenic, *LP* likely pathogenic

**Table 3:** Variants of Uncertain Significance found in 415 paediatric patients

P	S	GENE	INH	ZYG	VFS	XCI	Type	Ref Seq	Variant	dbSNP	Clinvar	MAF	CP	CADD	GERP	HSF
215	M	*AP1S2*	XL	HE	–	–	SYN	NM_003916.5	c.240G > A p.(Leu80Leu)	rs912444495	–	–	–	–	–	ESE site broken, New cryptic acceptor site
222	M	*ARID1B*	AD	HT	–	–	MIS	NM_001374828.1	c.1031C > T p.(Pro344Leu)	–	–	–	5	24.8	2.78	–
385	M	*ASH1L*	AD	HT	–	–	MIS	NM_018489.3	c.2950C > T p.(Arg984Cys)	rs548853139	–	0.00002005	10	27.2	5.16	–
		*FOXP1*	AD	HT	–	–	MIS	NM_001349338.3	c.1667 T > C p.(Ile556Thr)	rs751381124	–	0.000003976	7	24.3	6.13	–
19	M	*ASH1L*	AD	HT	–	–	MIS	NM_018489.3	c.6916C > T p.(Arg2306Trp)	rs142371619	–	7.311e-05	11	31	4.96	–
400	F	*ASH1L*	AD	HT	–	–	MIS	NM_018489.3	c.7543C > T p.(Arg2515Trp)	rs747531593	–	0.00001996	9	32	2.74	–
84	M	*ATRX*	XL	HE	MAT	MAT, 32:68	MIS	NM_000489.6	c.4196A > G p.(Glu1399Gly)	–	–	–	9	24.5	5.24	-
296	M	*ATRX*	XL	HE	MAT	MAT, 26:74	MIS	NM_000489.6	c.4407 T > A p.(Asp1469Glu)	–	–	–	8	22.5	2.46	–
359	M	*CASK*	XL	HE	MAT	random	MIS	NM_001367721.1	c.44G > A p.(Cys15Tyr)	–	–	–	5	23.9	4.88	–
127	M	*CASK*	XL	HE	MAT	MAT, 32:68	MIS	NM_001367721.1	c.2640C > A p.(Asp880Glu)	–	–	–	3	12.3	0.197	–
374	M	*CHD8*	AD	HT	–	–	MIS	NM_001170629.2	c.203C > A p.(Pro68His)	–	–	–	4	23.5	4.78	–
273	F	*CNTNAP2*	AD/AR	HT	PAT	–	MIS	NM_014141.6	c.511C > T p.(Arg171Cys)	rs375032955	VUS	4.47E-02	11	25.7	5.47	–
				HT	MAT	–	SYN	NM_014141.6	c.2517 T > C p.(Asn839 =)	rs143358892	LB	0.00007075	–	–	–	–
268	M	*CNTNAP2*	AD/AR	HT	–	–	MIS	NM_014141.6	c.2368C > A p.(Arg790Ser)	rs200089329	VUS	–	8	27	5.6	–
223	M	*DEAF1*	AD/AR	HT	–	–	SP?	NM_021008.4	c.805-31C > G p.?	–	–	–	–	–	–	Broken Branch Point
401	F	*DEAF1*	AD/AR	HT	-	–	MIS	NM_021008.4	c.908G > T p.(Arg303Leu)	–	VUS	–	9	28.6	3.65	–
		*KATNAL2*	AD	HT	–	–	MIS	NM_001387690.1	c.1084A > G p.(Arg362Gly)	–	–	–	12	29.6	4.8	–
330	F	*EHMT1*	AD	HT	healthy sister negative	–	MIS	NM_024757.5	c.103G > A p.(Asp35Asn)	rs371134699	VUS	0.00003081	9	25.3	5.41	–
170	M	*GRIA3*	XL	HE	MAT	MAT,74:26	MIS	NM_000828.5	c.2053G > A p.(Gly685Ser)	–	–	–	6	25.3	5:45	–
345	M	*GRIK2*	AD/AR	HT	–	–	MIS	NM_021956.5	c.205A > G p.(Thr69Ala)	–	VUS	8.154e-06	7	22	5.67	–
380	F	*GRIN2A*	AD	HT	–	–	MIS	NM_001134407.3	c.3163G > C p.(Glu1055Gln)	rs370107080	VUS	0.00001591	8	26.1	5.33	–
378	M	*GRIN2B*	AD	HT	–	–	MIS	NM_000834.5	c.2557G > A p.(Val853Ile)	rs201477697	–	–	4	26	5.76	–
179	F	*GRIN2B*	AD	HT	–	–	MIS	NM_000834.5	c.4259A > G p.(Asp1420Gly)	–	–	–	6	27	5.23	–
59	M	*IL1RAPL1*	XL	HE	MAT	–	MIS	NM_014271.4	c.735G > C p.(Leu245Phe)	–	–	–	4	21.2	4.14	–
160	F	*IQSEC2*	XL	HT	–	–	MIS	NM_001111125.3	c.322C > T p.(His108Tyr)	–	–	–	4	23.4	2.81	–
85	F	*IQSEC2*	XL	HT	–	98:2	SYN	NM_001111125.3	c.999G > A p.(Lys333 =)	–	–	–	–	–	–	Alteration of the WT Donor site
152	M	*IQSEC2*	XL	HE	MAT	MAT,67:33	MIS	NM_001111125.3	c.2976G > T p.(Leu992Phe)	–	–	–	5		2.21	-
279	M	*KDM5C*	XL	HE	MAT	MAT,71:29	MIS	NM_004187.5	c.835G > C p.(Gly279Arg)	–	VUS	–	2	12.88	1:28	–
353	M	*KDM5C*	XL	HE	–	–	MIS	NM_004187.5	c.1259 T > C p.(Leu420Pro)	–	–	–	12	29.7	5.85	–
413	F	*KIRREL3*	AD	HT	–	–	MIS	NM_032531.4	c.1639G > A p.(Ala547Thr)	–	–	0.000004013	7	24.8	5.46	–
388	M	*MECP2*	XL	HE	MAT	MAT,64:36	MIS	NM_004992.4	c.1231A > G p.(Ser411Gly)	–	–	–	3	19.81	4.22	–
174	M	*MED12*	XL	HE	MAT	MAT,76:24	MIS	NM_005120.3	c.1994C > G p.(Ser665Cys)	rs764981858	VUS	0.00001104	8	24.2	4.49	–
51	M	*MED12*	XL	HE	DN	–	SYN	NM_005120.3	c.3909C > T p.(Asp1303 =)	–	–	–				Altered ESE / ESS motifs ratio (-5)
155	F	*MED12*	XL	HT	–	–	MIS	NM_005120.3	c.4888G > A p.(Asp1630Asn)	–	VUS	–	4	23.2	4.15	–
283	M	*MED12*	XL	HE	MAT	MAT,60:40	MIS	NM_005120.3	c.5095C > G p.(Pro1699Ala)	–	–	–	10	24	4.17	–
390	M	*MED13L*	AD	HT	–	–	MIS	NM_015335.5	c.1367C > G p.(Ser456Cys)	–	–	–	6	22.5	5.76	–
234	M	*PPP2R5D*	AD	HT	-	–	NONFS	NM_006245.4	c.123_140del p.(Pro42_Gln47del)	–	–	–	–	–	–	–
21	F	*PPP2R5D*	AD	HT	–	–	MIS	NM_006245.4	c.1606A > T p.(Thr536Ser)	–	–	–	4	22.6	5.37	–
420	M	*PQBP1*	XL	HE	MAT	MAT,73:28	MIS	NM_005710.2	c.530G > A p.(Arg177His)	–	VUS	–	7	27	4.13	–
402	M	*PTCHD1*	XL	HE	–	–	MIS	NM_173495.3	c.605G > A p.(Arg202Gln)	rs771036286	–	0.000005453	5	22	3.99	–
149	M	*PTCHD1*	XL	HE	MAT	random	MIS	NM_173495.3	c.751C > T p.(Pro251Ser)	rs368662150	VUS	0.000005449	5	18.37	4.86	–
415	M	*PTCHD1*	XL	HE	MAT	MAT,37:63	MIS	NM_173495.3	c.1624A > G p.(Thr542Ala)	–	–	–	5	20.4	5.69	-
153	M	*SCN2A*	AD	HT	–	–	MIS	NM_001040142.2	c.2496C > A p.(Ser832Arg)	–	–	–	12	25.1	5.59	–
360	M	*SLC9A6*	XL	HE	–	–	MIS	NM_001042537.2	c.1034C > T p.(Thr345Ile)	-	–	–	8	27.9	5.35	–
111	M	*SYNGAP1*	AD	HT	–	–	MIS	NM_006772.3	c.1003C > T p.(Arg335Cys)	rs752399563	–	0.000003979	8	26.2	3.63	–
235	M	*TANC2*	AD	HT	–	–	MIS	NM_025185.4	c.1364A > G p.(Tyr455Cys)	rs376257499	–	0.00001070	11	25	5.23	–
369	M	*TANC2*	AD	HT	–	–	MIS	NM_025185.4	c.2978A > G p.(Gln993Arg)	–	–	–	8	26.8	5.78	–
325	M	*TRIO*	AD	HT	–	–	MIS	NM_007118.4	c.3641C > T p.(Ala1214Val)	rs373893038	–	0.008126	5	25.8	5.93	–
176	M	*TRIO*	AD	HT	–	–	MIS	NM_007118.4	c.5894G > A p.(Ser1965Asn)	–	–	–	4	23.3	5.48	–

**Abbreviations**:  *P* patient code, *S* sex, *INH* mode of inheritance, *ZYG* zygosity, *VFS* Variant Family Segregation, *FH* familial history, *Ref Seq* reference sequence, *MAF* minor allele frequency, *CP* consensus prediction among 12 computational tools provided by Annovar, *CADD* Combined Annotation Dependent Depletion score, *GERP* genomic evolutionary rate profiling score, *M* Male, *F* Female, *AD* autosomal dominant, *AR* autosomal recessive, *XL* X-linked, *HT* heterozygous, *HE* hemizygous, *MOS* mosaicism, *DN* de novo, *MAT* maternal, *PAT* paternal, *MIS* missense, *FS* frameshift, *P* pathogenic, *LP* likely pathogenic, *XCI* X-chromosome inactivation

*Notes:* For variants identified in X-linked genes, based on the X-inactivation analysis, the % of the active mutated allele in carrier female was reported.

**Table 4:** Mutated genes in the different phenotypic manifestations (ASD, epilepsy, Microcephaly, Macrocephaly, Hypotonia, and Ataxia). Some genes have been found mutated in individuals presenting phenotypic traits that have not been previously associated with these genes (highlighted in bold)

Clinical features	Affected individuals	Genes carrying pathogenic/likely pathogenic variants
Autistic traits	205	*ADNP, ARID1B, ASH1L, CHD8, DEAF1, DYRK1A, FOXP1, GRIN2A, MECP2, PHF21A, PTCHD1, RELN, SATB2, SHANK3, SLC6A1, SYNGAP1, WAC*
Epilepsy	84	*ANKRD11, CHD8, DYRK1A, EHMT1, GRIN2A, MBD5, MECP2, PHF21A, SLC6A1, SYNGAP1*
Microcephaly	45	***CHD8****, EHMT1, ****WAC****, ANKRD11, ****MED12****, CTNNB1, MECP2, ANKRD11, DYRK1A*
Macrocephaly	40	***ADNP****, FOXP1, GATAD2B, KDM5C, PPP2R5D*
Hypotonia	71	*ADNP, ****ANKRD11****, ARID1B, CTNNB1, FOXP1, KDM5C, MECP2, SATB2, WAC*
Ataxia	30	***CHD8****, DEAF1, MECP2, SYNGAP1, ****WAC***

The 49 VUS selected for 14% of our cases were found in 32 different genes (see Table 3). Further evidence will be required to confirm the association of these variants with the patient’s phenotype (see Table 3). During the prioritisation of these variants, significant consideration is given to predictions and conservation scores such as CADD and GERP + + , as well as to their frequency in the general population (e.g., gnomAD). Out of these 49 variants, 43 are missense, four are synonymous, one is intronic, and one is a non-frameshift deletion. Among the missense variants, 22 have a CADD score greater than or equal to 25, and 37 variants have a GERP + + score greater than 3 (see Table 3). Among the analysed variants, 32 are not found in the population database, and 18 have a significantly low frequency. Familial inheritance has been investigated in 16 cases, while the majority (*n* = 33) lack segregation analysis; one of the synonymous variants has been confirmed as de novo. Among the inherited variants, except for two heterozygous variants in *CNTNAP2* found in *trans*, the rest are mapped to X-linked genes and are maternally inherited. Most asymptomatic female carriers of X-linked gene variants showed X-inactivation favouring the wild-type allele. The four synonymous variants identified in *AP1S2*, *IQSEC2*, *MED12*, and *CNTNAP2*, and the deep intronic variant in *DEAF1*, are predicted to impact splicing mechanisms by HSF (see Table 3).

Four VUSs co-occurred with pathogenic variants in four individuals. In detail, the female proband (UniPD_0330) exhibited a heterozygous rare variant p.(Asp35Asn) in *EHMT1* in addition to a heterozygous *ANKRD11* p.(Lys635GlnfsTer26) pathogenic variant. In this case, the healthy sister did not carry either variant. In addition to the heterozygous frameshift deletion *p.*(Pro2383GlnfsTer47) in *CHD8*, the male proband (UniPD_0390), also presented the heterozygous variant in *MED13L* p.(Ser456Cys). In the UniPD_0170 we found the heterozygous frameshift variant p.(Asp826ArgfsTer11) in *SYNGAP1* and the hemizygous variant p.(Gly685Ser) in *GRIA3*. Finally, in UniPD_0388 we detected a novel missense variant in *MECP2* in hemizygous state and the heterozygous pathogenic variant p.(Asp8GlufsTer75) in *MBD5*. In two of these cases, UniPD_0330 and UniPD_0390, parent segregation was not possible due to adoption, resulting in uncertain significance in the interpretation of the missense variants in *EHMT1* and *MED13L*. However, the phenotypes observed in these two cases included clinical features associated with both the pathogenic and the VUS variants. In the other two cases, UniPD_0170 and UniPD_0388, the XCI analysis supports the pathogenicity of the variants in the X-linked genes, *MECP2* and *GRIA3* (see Table 3).

Finally, in 82 patients, we identified variants in 14 genes, such as *RELN*, *KATNAL2*, *MIB1*, associated with autism susceptibility, with low penetrance or with limited information about their involvement in NDDs. These variants were classified by Padova NDD laboratory as Risk Factors (see Supplementary Table S2).

#### Genotype–phenotype association: atypical findings

A systematic analysis by comparing patient phenotypes with HPO terms, revealed atypical genotype–phenotype cases where the observed clinical features did not fully align with the genetic alterations (see Table 4). One of the most frequent unexpected findings involved variations in head size. Specifically, patient UniPD_0129 with a frameshift variant in *ADNP* presented with macrocrania, despite the gene being primarily associated with skull anomalies such as plagiocephaly, trigonocephaly, and, less frequently, microcephaly. Conversely, three cases with pathogenic variants in *CHD8*, *WAC*, and *MED12* exhibited microcephaly, despite these genes typically not being associated with reduced head size. Case UniPD_0148 with the pathogenic *WAC* variant p.(Ser125Ter) showed microcephaly, which has been inconsistently reported in other *WAC* patients. Similar cases have been documented in the literature (Leonardi et al. 2020; Quental et al. 2022), although macrocephaly has also been reported in *WAC* patients (Lugtenberg et al. 2016; Uehara et al. 2018). This suggests that *WAC*-related NDDs may present a broader phenotypic spectrum than previously recognized.

Case UniPD_0210 with the p.(Ala1383Thr) variant in *MED12* also presented microcephaly. *MED12* variants are typically associated with X-linked syndromes featuring macrocephaly (Lujan-Fryns and Opitz-Kaveggia syndromes). Microcephaly has been rarely reported in certain Ohdo syndrome subtypes, including two siblings carrying the same variant found in our patient (Langley et al. 2015). This raises the possibility of distinct *MED12* variant-specific effects influencing cranial development.

In contrast, case UniPD_0062 harbored the truncating p.(Gln392Ter) variant in *CHD8*, which is predominantly linked to macrocephaly and ASD (OMIM#615,032) (Amberger et al. 2019). However, this patient exhibited microcephaly along with other atypical features, such as ataxia and digital anomalies, suggesting potential modifying factors or additional genetic contributors influencing the phenotype.

### Comparison with the CAGI5 dataset

The cohort of individuals selected for CAGI6 shows clinical characteristics similar to those of the population used in the previous CAGI5 edition (Aspromonte et al. 2019). Among patients with cognitive assessment, a higher distribution of individuals with mild to moderate ID is observed—specifically, 31% and 28% respectively—compared to 22% and 25.3% in the CAGI5 cohort (see Table 4). In the CAGI6 cohort, a lower percentage of patients had ASD or epilepsy, specifically 49% and 20% respectively, compared to 62% and 36.6% in the CAGI5 cohort. On the other hand, patients with macrocephaly are more represented in CAGI6, accounting for 10% compared to 7.3% in CAGI5.

The two cohorts slightly differ in the proportion of pathogenic variants we were able to identify, with 17,3% in CAGI5 and 14.5% in the CAGI6; while, the amount of variants with a likely although not established pathogenic role is higher in the CAGI6 (13%) compared to the previous one (10%).

As shown in Fig. 1, the number of genes with P and LP variants is more than doubled in CAGI6 compared to CAGI5. A substantial difference is also observed when comparing the mutated genes. In CAGI5, a particularly high mutation frequency was noted in *SHANK3, MECP2,* and *ANKRD11*, along with other genes carrying pathogenic variants that significantly contributed to defining the associated pathological conditions, such as *TRIO*. In CAGI6, *ANKRD11* and *MECP2* remained among the most frequently mutated genes, whereas *SHANK3* appeared less frequently, with only one pathogenic variant reported in this study (Table 2). The most frequently mutated genes in CAGI6 were completely absent in the 150 patients analyzed in CAGI5, including *ARID1B, CHD8,* and *ADNP*. In some genes, such as *KDM5C*, the presence of multiple mutations contributed to delineating a broader phenotypic spectrum (Leonardi et al. 2023).

## Discussion

### Genes with higher number of pathogenic variants

In nine genes (*ANKRD11, MECP2, ARID1B, CHD8, KDM5C, FOXP1, SYNGAP1, ADNP, SLC6A1*) we found more than two pathogenic variants. Importantly, we found multiple individuals with P/LP variants in *ASH1L* and *WAC* genes, recently established as definitive NDDs-related genes (see Supplementary Table S3) (Emanuela et al., 2020). Most of the variants we classified as pathogenic were predicted to result in truncated proteins and occurred in genes with strong evidence of dosage sensitivity (*ANKRD11, MECP2, ARID1B, FOXP1, SYNGAP1, ADNP,* and *WAC)* (see Supplementary Table S3). Truncating variants is the best-characterised class of variants due to their straightforward mechanism of action and prevalence in children with NDD (Firth et al. 2009). However, in some of these genes, we also identified pathogenic missense variants.

Although the impact of this class of variants on gene function is more challenging to interpret, resources that collect genetic data from control populations (e.g., gnomAD) and affected individuals (e.g., ClinVar) have been essential for establishing the deleteriousness of these variants. For instance, in patients exhibiting Rett phenotype (UniPD_0118, UniPD_0081), the identified *MECP2* missense variants were previously reported as known disease mutations in public databases, allowing us to classify them as pathogenic, even without segregation analysis (see Table 2). Additionally, in the case of *SLC6A1* missense variant (UniPD_0034), discovering that the known recurrent mutation in an affected individual was also present in the apparently healthy mother prompted us to investigate the family history further, revealing that the mother had experienced episodes of epilepsy during adolescence.

In some genes the identified missense variants were found in protein regions presenting clusters of pathogenic variants. Protein residues near or within clusters of pathogenic variants are more likely to be disease associated (Pérez-Palma et al. 2020). This evidence, particularly for newly identified variants, can be used as an additional criterion for variant interpretation, as “PM1” category of the ACMG’s guidelines. PM1 is defined as “variants located in a mutational hot spot and/or critical and well-established functional domain without benign variation”. Frequently, the mutation clusters align with structured domains of the protein that are crucial for its primary functions. This is the case of missense variants we identified in the *SLC6A1*, *ARID1B*, *CHD8*, *DEAF1*, *EHMT1*, and *PPP2R5D* genes. For instance, the three variants identified in the *SLC6A1* gene alter two functional elements of the γ-Aminobutyric acid (GABA) transporter (GAT1) which are enriched for patient over population variants with high decrease in GABA uptake compared to wild-type (Stefanski et al. 2023). The (p.(Trp285Cys) and p.(Gly307Arg) map to the TM6, forming the GABA binding pocket, and the p.(Gly362Arg) is located in the extracellular loop EL4. These findings were useful to confirm the pathogenicity for the newly identified variant p.Trp285Cys that may impact the protein folding disrupting the protein trafficking to the membrane (Motiwala et al. 2022) (see Supplementary Fig. S1).

In addition, patient variant clustering can be expected in functionally essential regions along the linear protein sequence. For instance, in the *ANKRD11* gene we identified the p.(Arg2536Trp) variant which is situated within a predicted highly charged alpha-helix region where other *ANKRD11* pathogenic variants have been observed in individuals with KBG syndrome (OMIM#148,050) (Boer et al. 2022) (Supplementary Figure S2). Missense variants of *ANKRD11* predominantly cluster in the RD2 repression domain of the protein's C-terminal region (amino acids 2369–2663), often involving arginine residues. These variants have been found to affect *ANKRD11* stability and potentially disrupt its proteasome degradation, leading to haploinsufficiency (Boer et al. 2022).

In other cases, the variant clustering is observed in larger regions with unknown functional roles. In these cases, collecting genetic data from different sources is extremely important. For instance, through collaborations with European groups working on NDDs we collected and described thirteen families with pathogenic *KDM5C* variants. This allowed us to observe a clustering of the variants at the C-terminal non-catalytic part of the protein, supporting the existence of distinct *KDM5C* regulatory functions that utilise enzymatic-independent molecular mechanisms (Leonardi et al. 2023).

In the case of *ASH1L* gene, we report in this study five missense variants. Although *ASH1L* variants are generally uncommon, most reported variants are of the truncating type (Stessman et al. 2017). Only a small number of de novo* ASH1L* missense variants associated with NDDs have been documented, and their functional impacts have not been experimentally investigated. We noticed that three of the detected variants (p.Arg984Cys, p.Asn1060Ser, p.Leu1298Arg), map in the N-terminal segment (aa: 900–1346) between two predicted AT-hook motifs suggesting the functional importance of this region (Table 2 and Table 3) (Supplementary Figure S3). A clustering of the missense variants in the *ASH1L* N-terminal portion has previously been observed (Liu et al. 2021).

### Variants of uncertain significance

Even in the selection of VUS, priority was given to factors such as pathogenicity predictions, involvement in functionally relevant regions, and clustering of pathogenic variants. For instance the p.(Arg303Leu) variant of *DEAF1* likely alters the nuclear localization signal (300-YKR**R**KKE-306) of the protein. This is consistent with functional studies showing that substitutions of Arg302 and Lys304 residues reduces the *DEAF1* protein cytoplasmic localization (Huggenvik et al. 1998) (see Supplementary Figure S4). Another example is the rare missense variant p.(Ser665Cys), which may affect a phosphorylation site in the Med12-LCEWAV domain. Additionally, the variant p.(Leu992Phe) in *IQSEC2* is located in a conserved sequence of the Pleckstrin homology domain (PH domain, 961–1078 aa), where other *IQSEC2* pathogenic variants cluster (see Supplementary Figure S5) (Shoubridge et al. 2022)**.** Overall, missense variants can have a range of effects on protein sequence and structure, including altering amino acid properties, disrupting protein folding, or affecting the stability of the protein. These changes can interfere with protein function, interaction with other molecules, or its localization within the cell. However, in most of the cases the mechanism underlying disease was found to cause a loss of function, particularly in genes that are highly sensitive to gene dosage. In such cases, even slight reductions in protein activity or expression are expected to lead to significant biological consequences, contributing to disease development.

### Unexpected genotype–phenotype correlations

Our findings underscore the complexity of genotype–phenotype correlations in NDDs. Although phenotype consistency is a critical factor in variant pathogenicity assessment, strict adherence to expected phenotypic presentations may lead to the exclusion of atypical cases that could enhance our understanding of gene-disease associations. The observed variations in head size across different genetic backgrounds suggest some genes may have broader phenotypic effects than previously recognized. In the case of *WAC*-related disorders, the presence of both microcephaly and macrocephaly across different individuals indicates a more variable phenotype. The increasing number of reported cases will likely refine our understanding of *WAC*'s role in neurodevelopment. Similarly, for *MED12*, our case as well as previously documented cases, supports the hypothesis that different variants within the gene may contribute to distinct phenotypic outcomes. This aligns with observations in other genes, such as *TRIO*, where opposing Rac1 modulations result in either microcephaly (OMIM#617,061) or macrocephaly (OMIM#618,825) (Barbosa et al. 2020).

The case of *CHD8*-associated microcephaly highlights the potential influence of genetic modifiers or additional pathogenic mechanisms. Given *CHD8*'s strong association with autism and macrocephaly, the presence of microcephaly in our case suggests either a unique consequence of the p.(Gln392Ter) variant or the impact of other contributing factors. Further investigations, including functional studies and genome-wide analyses, are needed to clarify these mechanisms.

Overall, these findings emphasize the challenges in predicting phenotypes based solely on genetic data, highlighting the need for integrative approaches considering additional genetic and environmental factors.

## Conclusions

This study presents the findings obtained by the application of a 74‐gene panel to perform DNA sequencing of 415 individuals with ID and ASD comorbidities. *ANKRD11* was found to be the most frequently mutated gene in this cohort, followed by *MECP2* and *ARID1B*. Further individuals have been found carrying de novo variants in *ASH1L* and *WAC* genes, for which limited cases have been described in literature. We identified atypical variants, such as a deep intronic variant in the *MED13L* gene and a mosaic missense variant in the *GRIN2A* gene. The functional impact and pathogenicity of these variants and others with uncertain significance were investigated based on previous knowledge, in silico evaluation and experimental evidence. Additionally, we discuss unexpected genotype–phenotype correlations observed in the study. In some cases, the phenotypes, in particular anomalies of the head size, did not fully align with the specific altered genes, challenging the establishment of pathogenicity. Of note the relationship between genotype and phenotype can be influenced by various factors, including genetic modifiers, environmental influences, and epigenetic modifications.

Overall, the study provides insights into the genetic landscape of NDDs and the identification of pathogenic variants, highlighting the complexity of genotype–phenotype correlations and the need for further investigation to understand the underlying mechanisms.

## Supplementary Information

Below is the link to the electronic supplementary material.

Supplementary file1 (DOCX 1459 KB)

Supplementary file2 (XLSX 209 KB)

## Data Availability

All data generated or analysed during this study are included in this published article and its Supplementary Information files.
