# Supplementary material for: Genetic variants and phenotypic data curated for the CAGI6 intellectual disability panel challenge
Source: Hum Genet. 2025 Feb 28;144(2-3):309–26. doi: 10.1007/s00439-025-02733-1 (PMC11976335; doi:10.1007/s00439-025-02733-1)
Supplement: Supplementary file 1 — Supplementary file1 (DOCX 1459 KB) [file 439_2025_2733_MOESM1_ESM.docx]

Human Genetics – CAGI6 Special Issue

Genetic Variants and Phenotypic Data Curated for the CAGI6 Intellectual Disability Panel Challenge

*Maria Cristina Aspromonte ^1,2^, Alessio Del Conte ^1^, Roberta Polli ^2,3^, Demetrio Baldo ^4^, Francesco Benedicenti ^5^, Elisa Bettella ^2,3^, Stefania Bigoni ^6^, Stefania Boni ^7^, Claudia Ciaccio ^8^, Stefano D’Arrigo ^8^, Ilaria Donati ^9^, Elisa Granocchio ^8^, Isabella Mammi ^10^, Donatella Milani ^11^, Susanna Negrin ^12^, Margherita Nosadini ^13^, Fiorenza Soli ^14^, Franco Stanzial ^5^, Licia Turolla ^4^, Damiano Piovesan ^1^, Silvio C.E. Tosatto ^1,15^, Alessandra Murgia ^2,3^, Emanuela Leonardi ^1,2,^**

*Correspondence: emanuela.leonardi@unipd.it (E.L.)

**SUPPLEMENTARY FIGURES**


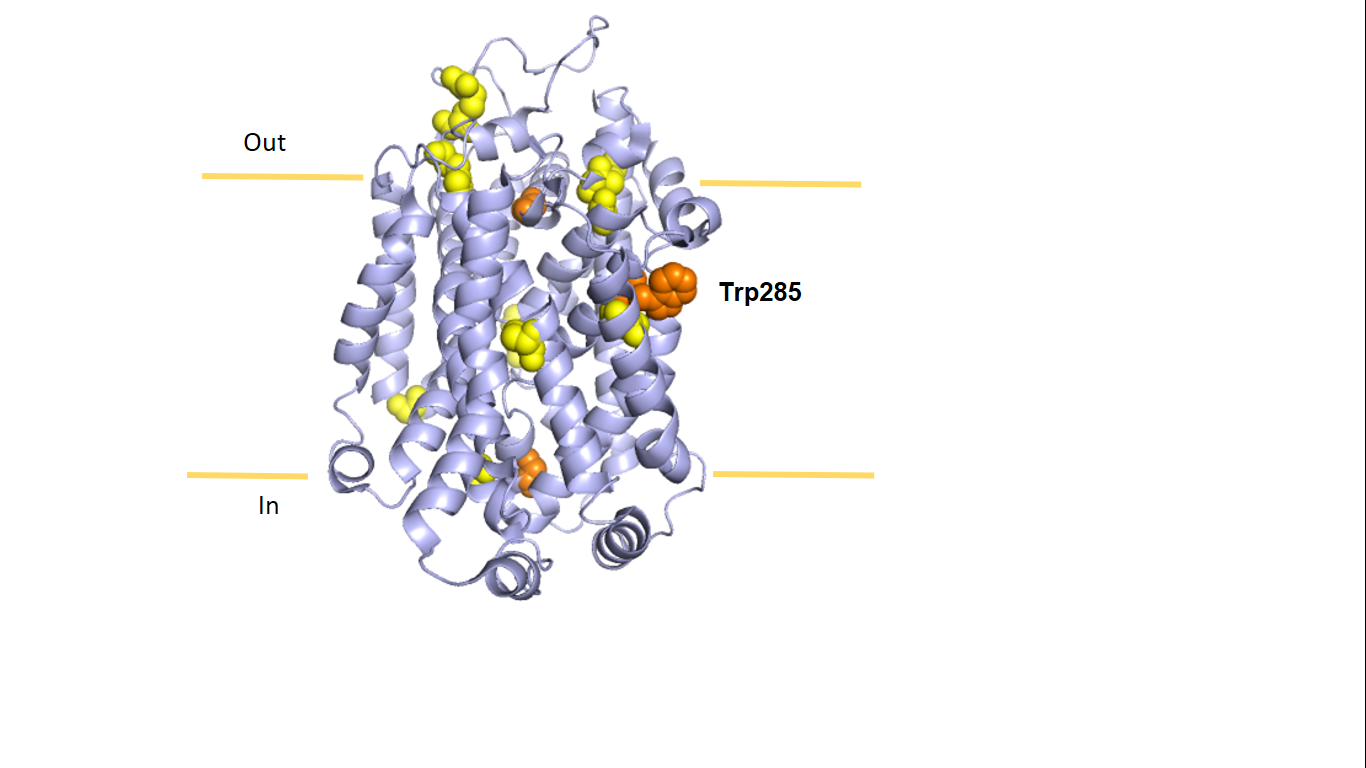


**Supplementary Figure S1. SLC6A1 variants mapping in the Transmembrane domain.** *SLC6A1 variants identified in our cohort (orange) and recurrent pathogenic variants reported in literature (yellow). The position of the mutated residues is shown as spheres in the in cryo-electron microscopy structure of the SLC6A1 Transmembrane domain (TM) (PDB code: 7SK2).*


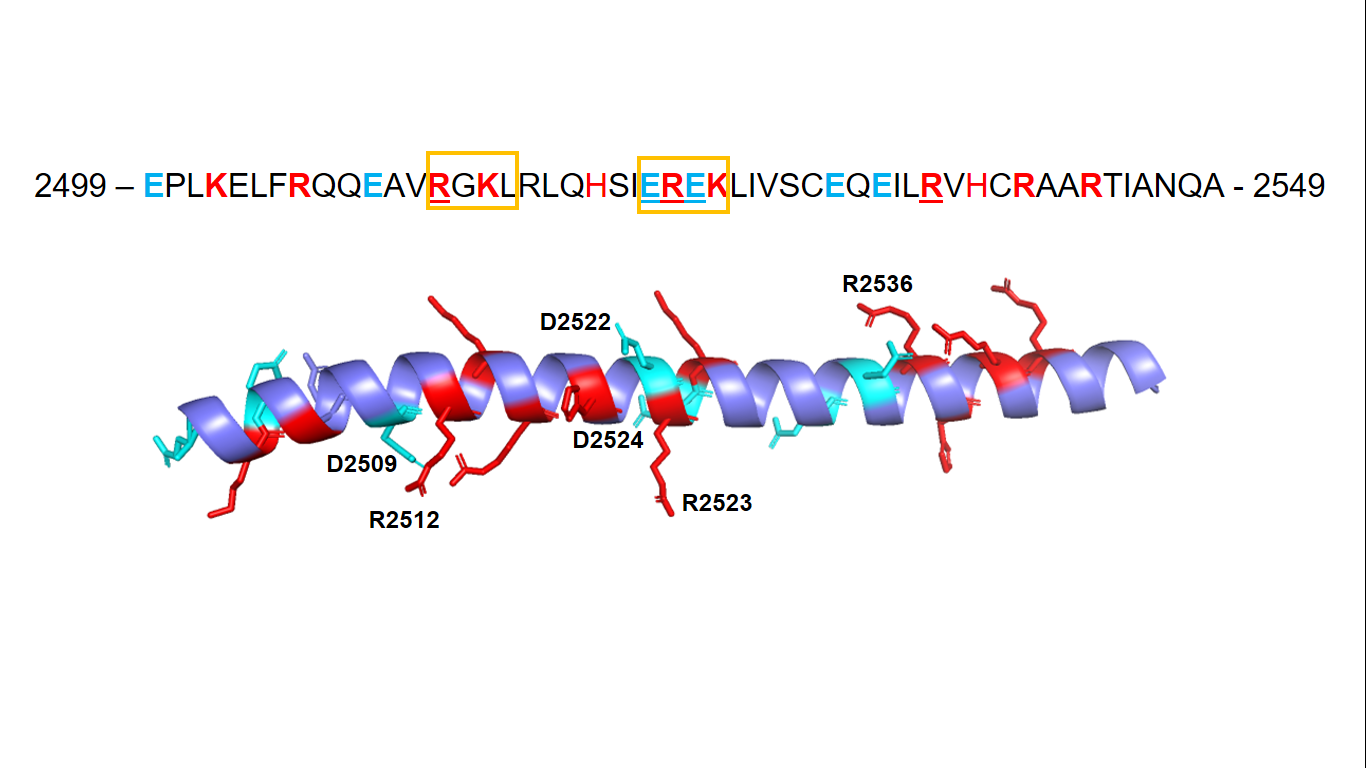


**Supplementary** [**Figure S**](https://docs.google.com/presentation/d/19bAxXi4kTz9i-9shIY11EGvYUKKaW1CB/edit)**2. The missense p.(Arg2536Trp) variant in *ANKRD11***. *Helix predicted by Alphafold spanning 2499-2549 residues. ANKRD11 amino acid sequence with the variants identified in affected individuals underlined. Positively and negatively charged residues are coloured in red and cyan, respectively. Proviz-predicted destruction motifs (D-boxes) are indicated in the sequence with orange boxes.*


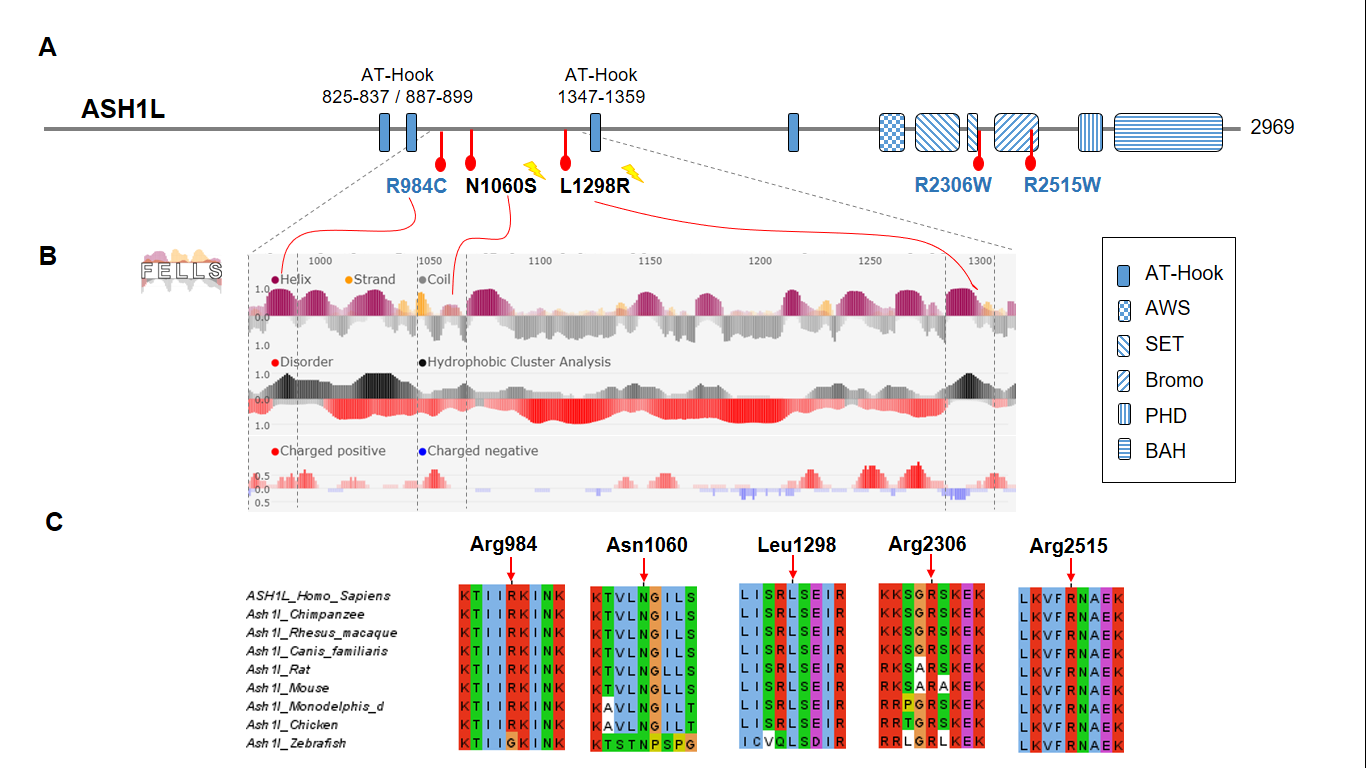


**Supplementary** [**Figure S3. ASH1L missense variants identified in this study**](https://docs.google.com/presentation/d/10qbPc4_rTccU-PA8Q0zWMlKcnKqUhXVQ/edit)*. (A) Domain architecture of ASH1L indicating locations of the identified missense variants. Three variants map in the N-terminal region between two predicted AT-hook motifs. A flash indicates de novo variants. Domains are indicated according to InterPro: AT-Hook, DNA binding motifs; AWS (Associated With SET) (aa 2091-2143); SET domain (aa 2145-2285); Bromo (aa 2442-2552); Zinc-finger, PHD type (aa 2587-2629); BAH (Bromo adjacent homology) (aa 2661-2798). (B) Visualization of secondary structure, intrinsic disorder and charged clusters predicted with FELSS [http://old.protein.bio.unipd.it/fells/]. Asn1060 maps in a hydrophobic region preceded by a cluster of positively charged residues; Leu1298 is predicted to form an amphipathic hydrophobic helix. (C) Asn1060 and Leu1298 residues are conserved among orthologous sequences. Multiple sequence alignment of ASH1L orthologs around the mutated positions. The sequences are coloured by clustal colour scheme with Jalview.*


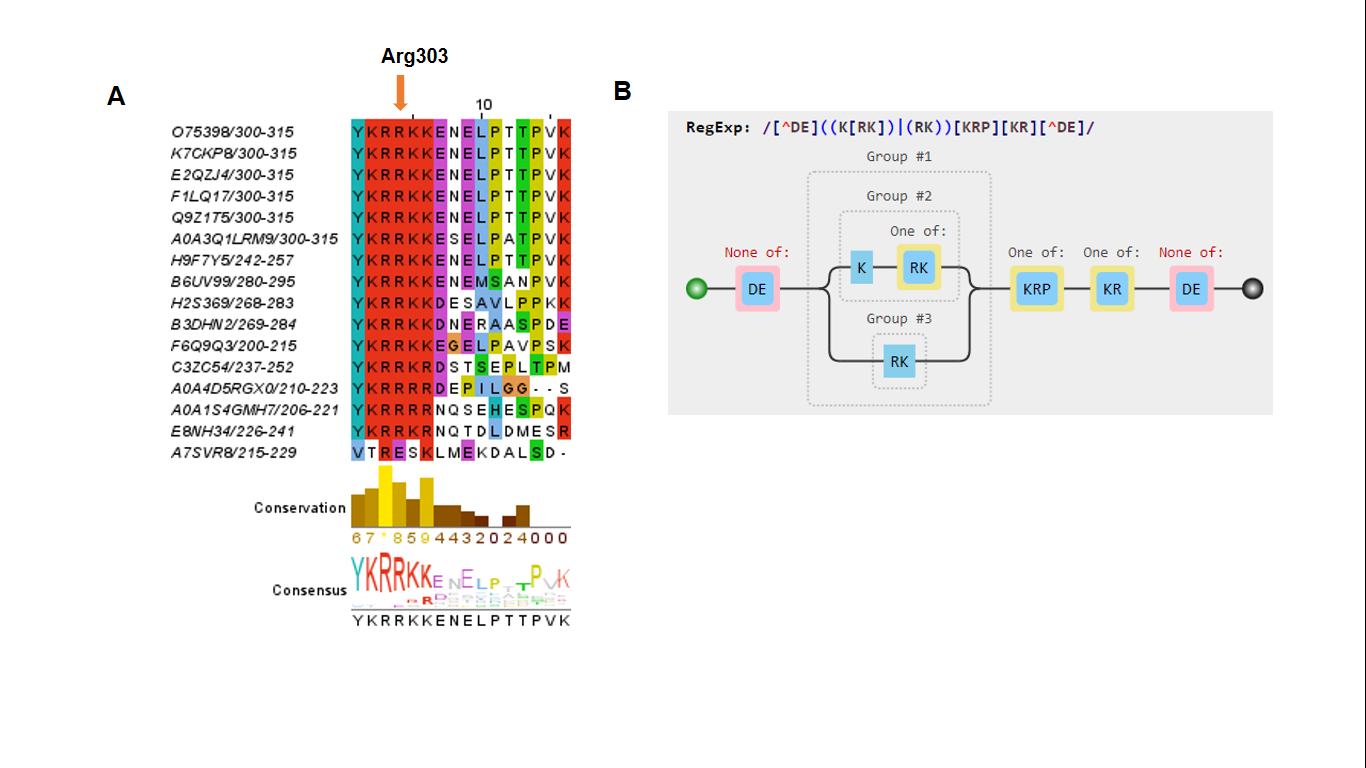


**Supplementary Figure S4. The missense variant p.(Arg303Leu) detected in DEAF1.** *(A) DEAF1 p.Arg303 residue and the entire Nuclear Localization Signal (300-YKRRKKE-306) are conserved among orthologous sequences. Sequences are coloured according to clustal color scheme with Jalview. (B) Sequence pattern of the Nuclear Localization Signal according to ELM.*


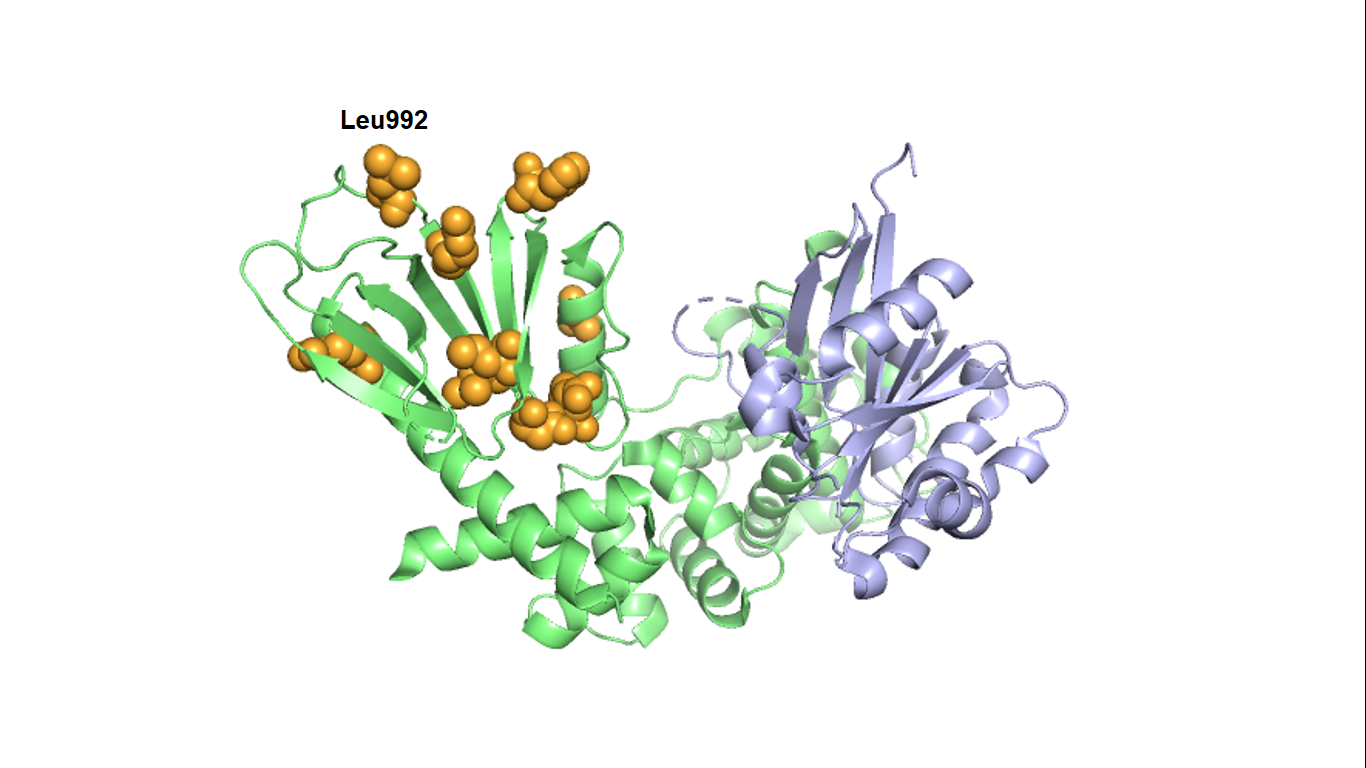


**Supplementary** [**Figure**](https://docs.google.com/presentation/d/13ABA81YvAnfKgRTIEYz1kLsPU4z2GqBe/edit) **S5. IQSEC2 variants in the PH domains.** *Mutated positions identified in our cohort and reported in literature are indicated as orange spheres and mapped in the crystal structure of the Sec7-PH domains of IQSEC2 (limegreen) in complex with the small GTPase Arf1 (lightblue) (PDB 6FAE). The position of the variant identified p.(Leu992Phe) in our cohort is indicated.*
